# Spinal HMGB1 participates in the early stages of paclitaxel-induced neuropathic pain via microglial TLR4 and RAGE activation

**DOI:** 10.3389/fimmu.2024.1303937

**Published:** 2024-02-07

**Authors:** Thamyris Reis Moraes, Flavio Protasio Veras, Angel Roberto Barchuk, Ester Siqueira Caixeta Nogueira, Alexandre Kanashiro, Giovane Galdino

**Affiliations:** ^1^ Pain Neuroimmunobiology Laboratory, Institute of Motricity Sciences, Federal University of Alfenas, Alfenas, Brazil; ^2^ Integrative Animal Biology Laboratory, Institute of Biomedical Sciences, Federal University of Alfenas, Alfenas, Brazil; ^3^ Department of Cellular and Developmental Biology, Federal University of Alfenas, Alfenas, Brazil; ^4^ Department of Dermatology, University of Wisconsin-Madison, Madison, WI, United States

**Keywords:** HMGB1, TLR4, RAGE, glial cells, neuropathic pain, chemotherapy

## Abstract

**Introduction:**

Chemotherapy-induced neuropathic pain (CINP) is one of the main adverse effects of chemotherapy treatment. At the spinal level, CINP modulation involves glial cells that upregulate Toll-like receptor 4 (TLR4) and signaling pathways, which can be activated by pro-inflammatory mediators as the high mobility group box-1 (HMGB1).

**Objective:**

To evaluate the spinal role of HMGB1 in the paclitaxel-induced neuropathic pain via receptor for advanced glycation end products (RAGE) and TLR4 activation expressed in glial cells.

**Methods:**

Male C57BL/6 Wild type and TLR4 deficient mice were used in the paclitaxel-induced neuropathic pain model. The nociceptive threshold was measured using the von Frey filament test. In addition, recombinant HMGB1 was intrathecally (i.t.) injected to confirm its nociceptive potential. To evaluate the spinal participation of RAGE, TLR4, NF-kB, microglia, astrocytes, and MAPK p38 in HMGB1-mediated nociceptive effect during neuropathic pain and recombinant HMGB1-induced nociception, the drugs FPS-ZM1, LPS-RS, PDTC, minocycline, fluorocitrate, and SML0543 were respectively administrated by i.t. rout. Microglia, astrocytes, glial cells, RAGE, and TLR4 protein expression were analyzed by Western blot. ELISA immunoassay was also used to assess HMGB1, IL-1β, and TNF-α spinal levels.

**Results:**

The pharmacological experiments demonstrated that spinal RAGE, TLR4, microglia, astrocytes, as well as MAPK p38 and NF-kB signaling are involved with HMGB1-induced nociception and paclitaxel-induced neuropathic pain. Furthermore, HMGB1 spinal levels were increased during the early stages of neuropathic pain and associated with RAGE, TLR4 and microglial activation. RAGE and TLR4 blockade decreased spinal levels of pro-inflammatory cytokines during neuropathic pain.

**Conclusion:**

Taken together, our findings indicate that HMGB1 may be released during the early stages of paclitaxel-induced neuropathic pain. This molecule activates RAGE and TLR4 receptors in spinal microglia, upregulating pro-inflammatory cytokines that may contribute to neuropathic pain.

## 1 Introduction

Chemotherapy-induced neuropathy is one of the main causes of pain in cancer patients. It is an adverse effect of chemotherapeutic agents with a dose-dependent effect, which affects about 68% of patients in the first month of treatment (1, 2). The increase in cancer incidence, survival and cure of patients has increased its prevalence, since common pharmacological approaches are ineffective, treatment discontinuation may occur (3).

Paclitaxel (PCT) is a chemotherapeutic agent that interrupts cellular mitosis by stabilizing the microtubules system, resulting in apoptosis-mediated cell death (4). However, cell cycle disruption by this chemotherapeutic agent is not limited to cancer cells, and neurons are highly susceptible to these effects (4). Thus, the mechanisms related to PCT-induced peripheral neuropathy include apoptosis, cell membrane remodeling, changes in neuronal ion channels, mitochondrial dysfunctions, and demyelination that impair the axonal transport system, leading to neuronal death (2, 5).

Following chemotherapy-induced nerve injury, several pro-inflammatory mediators are released, including damage-associated molecular patterns (DAMPs), which activate spinal glial cells by binding to receptors present on these cells, triggering intracellular signalization pathways that result in the production of cytokines that favor central pain sensitization (6, 7).

High mobility group box-1 (HMGB1), a structural cofactor that regulates transcriptional activities and gene expression in mammalian cells (6), may be an important mediator involved in pain sensitization. HMGB1 is released during cellular necrosis and can be activated by some mediators, such as macrophages and glial cells; thus acting as a DAMP (8). HMGB1 via receptor for advanced glycation end products (RAGE) and Toll-like receptor 4 (TLR4) activation has shown an important nociceptive role (9); however, few mechanisms have been investigated regarding this process.

There is limited evidence indicating that after RAGE and TLR4 activation, HMGB1 activates intracellular pathways such as c-Jun N-Terminal Kinase (JNK) and nuclear factor kappa B (NF-κB) (10), upregulating pro-inflammatory cytokines such as interferon-gamma (IFN-γ), interleukin-6 (IL-6), tumoral necrosis factor-α (TNF-α), and interleukin-1β (IL-1β); which are involved in the genesis of neuropathic pain (6, 11, 12). However, most of these studies were restricted to peripheral investigation. Thus, the present study aimed to investigate the participation of RAGE and TLR4 receptors activated by HMGB1 in spinal glial cells during PCT-induced neuropathic pain, as well as the intracellular mechanisms that participate in this process.

## 2 Materials and methods

### 2.1 Mice

All experiments used male C57BL/6 Wild type and TLR4 deficient mice (7-8 weeks old). The animals belong to the vivarium of the Federal University of Alfenas or the University of São Paulo. For the experiments, they were kept in boxes with 6 animals, with water and food until libido and maintained at a 12 h dark-light cycle and at a temperature that varied between 22 and 24°C with a relative air humidity at 50± 5%. The study was previously approved by the Ethics Committee for the Use of Animals of the Federal University of Alfenas (protocol number 52/2018) and was conducted in accordance with the IASP Guidelines for the Use of Animals in Research (13).

### 2.2 Microglial cell culture and treatment

C8-B4 mouse microglial cell lines were kindly donated by Prof. Niels Saraiva Camara from the University of Sao Paulo, Brazil, previously purchased from the American Type Culture Collection (ATCC, Manassas, VA, USA). The cells were cultured in DMEM media, supplemented with 10% heat-inactivated fetal bovine serum and 1% Penicillin–Streptomycin at 37°C in a humidified 5% CO2 incubator. To perform the experiments, cells were plated in 24-well plates at a concentration of 2x10^5^ per well. After two days of cultivation, cells were stimulated with LPS (1 µg/mL), HMGB1 (50 ng/ml) diluted in complete medium.

### 2.3 Mechanical nociceptive threshold measurement

To assess the nociceptive threshold, the von Frey filament test (Stoeling, Wood Dale, IL, USA) was used. For this test, the animals were acclimatized during 30 min in dark glass boxes, which were placed under a metal platform. The floor of the glass boxes is composed by small metal grids that allow the filaments to access the evaluated paws of each animal. After that, different thicknesses of filaments (0.07, 0.16, 0.4, 0.6, 1.0, 1.4, and 2.0 grams) were applied to the plantar surface of right paw of each animal, and the mean of pressure values corresponding to three withdrawals was recorded as the nociceptive threshold (14).

### 2.4 Paclitaxel-induced neuropathic pain model

The paclitaxel-induced neuropathic pain model used in the present study was adapted from Sekiguchi et al. (15), in which PCT (1 mg/kg, Sigma-Aldrich, MO, USA) was administered intraperitoneally, for 4 alternating days.

### 2.5 Drugs

In this study the following substances were used: Paclitaxel (PCT, Cayman Chemical Company, USA), a chemotherapeutic agent (15); FPS-ZM1 (Cayman Chemical Company, USA), a RAGE antagonist at doses of 25 μg, 50 μg, and 100 μg (16); LPS-RS (Invivogen, USA), a TLR4 antagonist at doses of 0.5 μg and 1 μg (14); Minocycline (Sigma-Aldrich, USA), a microglia inhibitor at doses of 5 μg and 10 μg (17); Fluorocitrate (Invivogen, USA), an astrocyte inhibitor at doses of 150 pmol and 300 pmol (14); SML0543 (Sigma-Aldrich, USA), p38 MAPK pathway inhibitor at doses of 1.5 nmol and 3 nmol (18); PDTC (Sigma-Aldrich, USA), a NF-kB inhibitor at doses of 30 μg and 60 μg and administered i.t. (19), and recombinant HMGB1 protein (Sigma-Aldrich, USA), at doses of 50 ng and 100 ng (20). PCT, FPS-ZM1 and SML0543 were diluted in sterile saline and DMSO (2%), and the other drugs were diluted in saline.

### 2.6 Injections

#### 2.6.1 Intrathecal

Firstly, the animals were anesthetized with isoflurate (2%) and positioned in prone position. Each drug used in the study, with the exception of PCT, was injected between the L5–L6 intervertebral segments, in a volume of 5 µL using a 10 µL syringe (Model 701N, Hamilton, USA) (21).

#### 2.6.2 Intraperitoneal

During intraperitoneal injections of PCT, the animals were carefully held upside down by one of the experimenter’s hands and with the other hand, using a 25-gauge needle, PCT in a 10 ml/Kg volume was given into the left lower quadrant (22).

### 2.7 Experimental design

**Figure 1:**
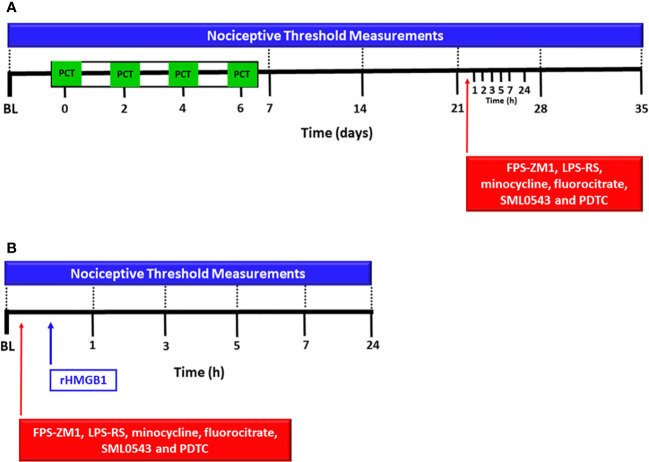
Experimental protocol for evaluation of spinal involvement of TLR4, RAGE, microglia, astrocytes, MAPK p38 and NF-kB in the nociception induced by paclitaxel or HMGB1. The baseline latency (BL) of the nociceptive threshold of each mouse was firstly measured using the von Frey filament test, and paclitaxel [PCT, **(A)**] or rHMGB1 **(B)** were subsequently injected. PCT was i.p. administered on 4 alternate days and rHMGB1 was administered in a single intrathecal injection. Other nociceptive threshold measurements were performed on the 7th, 14th, 21st, 28th and 35th day of neuropathic pain. After 15 days of PCT treatment (21st day of neuropathic pain) or rHMGB1 injection, respective antagonists or inhibitors FPS-ZM1, LPS-RS, minocycline, fluorocitrate, SML0543 and PDTC, were intrathecally administered, and new measurements of nociceptive threshold were taken at 1, 3, 5, 7, and 24 hours after each injection.

Initially, baseline of nociceptive threshold was measured. Then, PCT was intraperitoneally administered on 4 alternate days from baseline (days 0, 2, 4, and 6). The nociceptive threshold was also measured 7, 14, 21, 28 and 35 days after baseline measurement (**Figure 1A**). After evaluating PCT-induced nociceptive effect, complementary experiments were performed in order to investigate the spinal participation of RAGE, TLR4, microglia, astrocytes, MAPK p38 and NF-kB in neuropathic pain. For this, 21 days after baseline nociceptive threshold measurement (15 days after the last PCT injection), the respective antagonists or inhibitors FPS-ZM1, LPS-RS, minocycline, fluorocitrate, SML0543, and PDTC were intrathecally (i.t.) administered, and the nociceptive threshold was measured again 1, 3, 5, 7, and 24h after each injection. The twenty-first day was chosen because it was a period in which neuropathic pain was established and the nociceptive threshold had already reached a nociceptive peak.

Thus, the groups used were (n= 6 mice/group): PCT + FPS-ZM1 (25 µg/5 µL), PCT + FPS-ZM1 (50 µg/5 µL), and PCT + FPS-ZM1 (100 µg/5 µL), composed of mice with neuropathic pain pretreated with the RAGE antagonist FPS-ZM1; PCT + LPS-RS (500 ng/5 µL) and PCT + LPS-RS (1 µg/5 µL), composed of animals with neuropathic pain pretreated with the TLR4 antagonist LPS-RS; PCT + Minocycline (5 µg/5 µL) and PCT + Minocycline (10 µg/5 µL), composed of mice with neuropathic pain pretreated with the microglia inhibitor minocycline; PCT + Fluorocitrate (150 pmol/5 µL) and PCT + Fluorocitrate (300 pmol/5 µL), composed of mice with neuropathic pain pretreated with the astrocytes inhibitor fluorocitrate; PCT + SML0543 (1.5 nmol/5 µL) and PCT + SML0543 (3 nmol/5 µL), composed of mice with neuropathic pain pretreated with the MAPK p38 inhibitor SML0543; PCT + PDTC (30 µg/5 µL) and PCT + PDTC (60 µg/5 µL), composed of mice with neuropathic pain pretreated with the NF-kB inhibitor PDTC. The nociceptive threshold in mice with neuropathic pain treated with the vehicle of these substances was also evaluated.

In order to investigate the spinal nociceptive effect of HMGB1, as well as RAGE, TLR4, microglia, astrocytes, MAPK p38 and NF-kB involvement in this process; the baseline latency was firstly measured. Then, recombinant HMGB1 (rHMGB1) or vehicle was administered intrathecally, and additional nociceptive threshold measurements were obtained after 1, 3, 5, and 24h (**Figure 1B**). The inhibitors or antagonists were administered intrathecally 20 min before rHMGB1 administration. The groups used were the following (n = 6 mice/group): rHMGB1 (50 ng/5 µL) and (100 ng/5 µL), composed of animals receiving i.t. rHMGB1 administration; FPS-ZM1 (100 ng/5 µL) + rHMGB1 (50 ng/5 µL), consisting of animals pretreated with the RAGE antagonist FPS-ZM1 that received rHMGB1; LPS-RS (1 µg/5 µL) + rHMGB1 (50 ng/5 µL), composed of animals pretreated with the TLR4 antagonist LPS-RS followed by rHMGB1 injection; TLR4^-/-^ + rHMGB1 (50 ng/5 µL), composed of TLR4^-/-^ knockout animals receiving i.t. rHMGB1 administration; Minocycline (10 µg/5 µL) + rHMGB1 (50 ng/5 µL), composed of animals pretreated with the microglia inhibitor minocycline followed by rHMGB1 i.t. administration; Fluorocitrate (300 pmol/5 µL) + rHMGB1 (50 ng/5 µL), composed of animals pretreated with the astrocytes inhibitor fluorocitrate followed by i.t. rHMGB1 administration; SML0543 (3 nmol/5 µL) + rHMGB1 (50 ng/5 µL), composed of animals pretreated with the MAPK p38 inhibitor SML0543 followed by i.t. rHMGB1 administration; PDTC (60 µg/5 µL) + rHMGB1 (50 ng/5 µL), composed of animals pretreated with the NF-kB inhibitor PDTC followed by i.t. rHMGB1 administration (**Figure 1B**). Animals with neuropathic pain treated with the vehicle of these substances were also evaluated. All groups described above are represented in **Table 1**.

**Table 1:** Experimental groups used in the study.

PCT-induced neuropathic pain groups	Concentrations	Description
PCT + FPS-ZM1	25, 50 e 100 µg/5 µL	mice with neuropathic pain pretreated with the RAGE antagonist
PCT + LPS-RS	00 ng and 1 µg/5 µL	mice with neuropathic pain pretreated with the TLR4 antagonist
PCT + Minocycline	5 and 10 µg/5 µL	mice with neuropathic pain pretreated with the microglia inhibitor minocycline
PCT + Fluorocitrate	150 and 300 pmol/5 µL	mice with neuropathic pain pretreated with the astrocytes inhibitor fluorocitrate
PCT + SML0543	1.5 and 3 nmol/5 µL	mice with neuropathic pain pretreated with the MAPK p38 inhibitor SML0543
PCT + PDTC	30 and 60 µg/5 µL	mice with neuropathic pain pretreated with the NF-kB inhibitor PDTC

HMGB1-induced nociception groups		
rHMGB1	50 ng/5 µL	mice that received recombinant HMGB1
FPS-ZM1 + rHMGB1	100 ng/5 µL*	mice pretreated with FPS-ZM1 and that received rHMGB1
LPS-RS + rHMGB1	1 µg/5 µL*	mice pretreated with LPS-RS and that received rHMGB1
Minocycline + rHMGB1	10 µg/5 µL*	mice pretreated with minocycline and that received rHMGB1
Fluorocitrate + rHMGB1	300 pmol/5 µL*	mice pretreated with fluorocitrate and that received rHMGB1
SML0543 + rHMGB1	3 nmol/5 µL*	mice pretreated with SML0543 and that received rHMGB1
PDTC 60 µg/5 µL + rHMGB1	60 µg/5 µL*	mice pretreated with PDTC and that received rHMGB1
TLR4^-/-^ + rHMGB1		TLR4 knockout mice that received rHMGB1

Groups sizes and power analyses were calculated using G*Power software. In the t test, the effect size was first calculated from means and standard deviation. Then, the effect size, α, and sample size groups were used to perform power analysis. In the F test, the effect size was first calculated from variance explained by a special effect and variance within groups. Then, the effect size, α, total sample size, and number of groups were used to perform power analysis.

#### 2.7.1 HMGB1, RAGE, TLR4, microglia, and astrocytes expression during PCT-induced neuropathic pain

The spinal HMGB1, RAGE, TLR4, microglia, and astrocytes protein levels during chemotherapy-induced neuropathic pain were analyzed by Western blot. To evaluate HMGB1 protein levels, spinal cord samples (lumbar segments L4–L6) from PCT- (1 mg/kg per injection) or vehicle-treated mice were collected after the first day of treatment; as well as 7, 14 and 21 days after the last PCT or vehicle administration. RAGE and TLR4 spinal cord levels were evaluated 14 and 21 days after the last PCT or vehicle administration. In addition, the microglia marker Iba1 and the astrocytes marker GFAP protein levels were evaluated in mice with neuropathic pain and health controls, pretreated or not with FPS-ZM1 (100 ng/5 µL, i.t.) and LPS-RS (1 ng/5 µL, i.t.), 21 days after the PCT or vehicle treatment.

After euthanizing the animals with isoflurane (5%), spinal cord tissues (lumbar segments L5-L6) were collected and added to microtubes containing RIPA buffer and protease inhibitors cocktail (Sigma-Aldrich, USA). Immediately, the tissues were homogenized and centrifuged. Then, the supernatant was collected and the proteins from each sample were measured using the Bradford method, separated by electrophoresis and transferred to a nitrocellulose membrane through a semi-dry system (transblot turbo, Bio-Rad, USA). After washing with TBS-T and blocking with 5% nonfat milk for 2h, each membrane was incubated with the respective primary antibodies: mouse anti-HMGB1 (1:500, Cayman Chemical Company, USA), mouse anti-RAGE (1:500, Santa Cruz, USA), rabbit anti-TLR4 (1:400, Cell Signaling, USA), rabbit anti-Iba1 (1∶500, Wako, Japan), mouse anti-GFAP (1:500, Boster Biological Technology, USA), and mouse anti-β-actin (1:5000, Sigma-Aldrich, USA) antibodies. After 3 washes, the membranes were incubated at room temperature with secondary antibodies, such as anti-rabbit IgG HRP (1:2000, Santa Cruz, USA), and anti-mouse IgG HRP (1:2000, Abcam, UK). Then, the membranes were washed again and a chemiluminescence kit was applied (Bio-Rad, USA) was applied for 3min. The images of the bands were captured and analyzed by the ChemiDoc MP Imaging System (Bio-Rad, USA), and the intensities were quantified by Image Lab software (Bio-Rad, USA). The intensity of band was normalized by β-actin protein expression. The data were expressed as fold changes compared to the control group.

### 2.8 HMGB1, IL-1β, IL-10 and TNF-α quantification

In order to investigate the HMGB1, IL-1β and TNF-α spinal levels in spinal cord during PCT-induced neuropathic pain, and IL-10 and TNF-α levels after microglia stimulation *in vitro*, the ELISA assay was performed. For this, spinal cord samples (segments L4–L6) were collected at the on day 21 days of neuropathic pain or control in mice non-treated and mice pretreated and non-treated with FPS-ZM1 (100 ng/5 µL) and LPS-RS (1 ng/5 µL). In *in vitro* experiments, the medium was collected for dosing after 12 hours of stimulation with LPS (1 µg/mL), HMGB1 (50 ng/ml) or vehicle. HMGB1, IL-1β, IL-10 and TNF-α levels were determined by specific kit (Prepotech, Cranbury, NJ, USA). After that, 200 mg of each sample was transferred to microtube containing 1 mL of PBS for subsequent homogenization for 10 min at 3000 rpm at 4°C. Finally, the supernatants are transferred to 96-well microplates for subsequent analysis and detection of cytokines using a microplate reader (ELX800, BioTek, VT, USA) and the Gen5 software (BioTek, VT, USA).

### 2.9 Flow-cytometric analysis

Twelve hours after LPS or HMGB1 stimulation, expressions of cell surface antigens CD45 (Elabscience, TX, USA) and Annexin V (SouthernBiotech, AL, USA) were analyzed by flow cytometry using a Guava® easyCyte (Millipore, MO, USA). For experiments, cells were detached by 0.25% trypsin treatment and resuspended in PBS containing 0.5% bovine serum albumin and 2 mM EDTA. The cells were stained with FITC-labeled anti-CD45 antibody (eBioscience, San Diego, CA, USA) or Annexin V for 30 min at 4 °C. The MFI was measured in a total of 10.000-counted cells using the FlowJo software (Tree Star, CA, USA).

### 2.10 Statistical analysis

The results of analysis were shown as mean and standard error of the mean (mean ± S.E.M.). For the behaviors experiments, was used the Two-way ANOVA analysis, and the One-way ANOVA analysis was used for ELISA and Western blot results. The Bonferroni’s *post-hoc*-test was used followed both ANOVA analysis. P < 0.05 was considered the minimum level of significance. Statistical analysis and graphs were performed using the GraphPad Prism 5 software (GraphPad Software, USA).

## 3 Results

### 3.1 TLR4 and RAGE participation in PCT and HMGB1-induced mechanical allodynia

**Figure 2:**
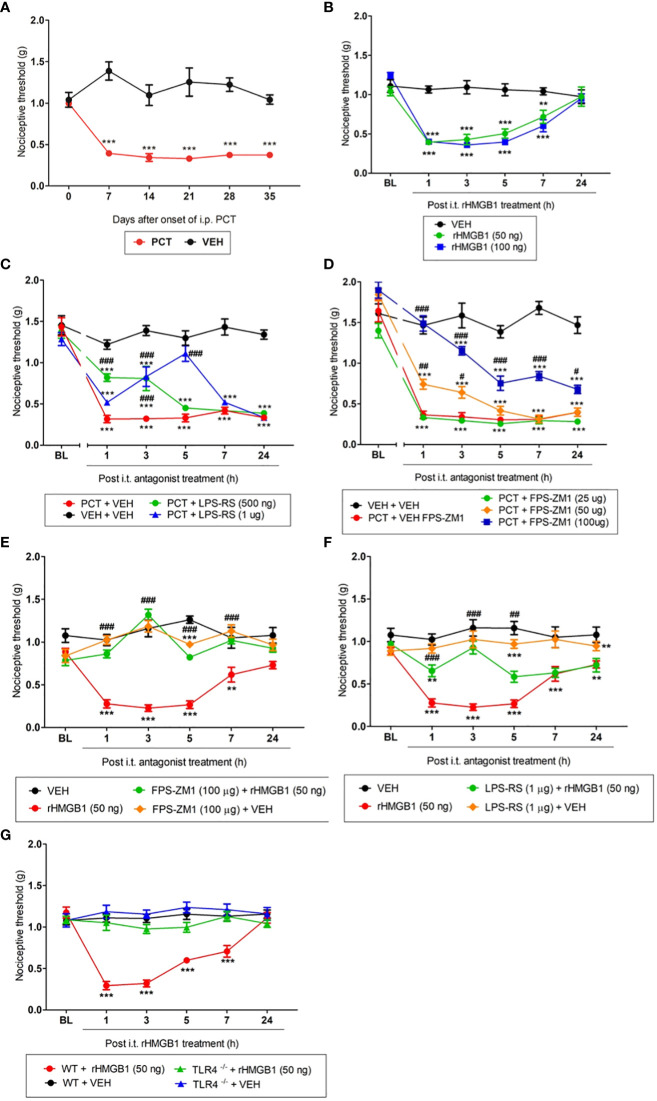
Involvement of the TLR4 and RAGE during paclitaxel (PCT) and HMGB1-induced mechanical allodynia. Mice that received PCT treatment (i.p.) **(A)** or rHMGB1 i.t. injection **(B)** presented mechanical allodynia. This effect was reversed by specific TLR4 **(C, F)** and RAGE **(D, E)** antagonists, respectively, administered 15 days after the end of PCT treatment (21st day) or three hours after rHMGB1. **(G)** HMGB1-induced mechanical allodynia was inhibited in TLR4 deficient mice (TLR-/-). Data are expressed as the mean ± SEM of 6 animals per group. **P < 0.01 and ***P < 0.001 indicate statistical difference compared to animals that received saline (Vehicle: VEH); #P < 0.05, ##P < 0.01 and ###P < 0.001 indicate statistical difference compared to PCT or rHMGB1 groups. Two-way ANOVA followed by the Bonferroni test (factors: time and treatment). BL, baseline latency.

First, to evaluate PCT effect on the nociceptive threshold in our experimental model, mechanical allodynia was measured 1, 7, 14, 21, 28, and 35 days after PCT or vehicle treatment. Thus, **Figure 2A** shows a significant (P < 0.001; F1,10 = 11.64) mechanical allodynia after every day of measurements, indicating that neuropathic pain was successfully established. rHMGB1-induced nociceptive effect was also evaluated through its intrathecal injection. Mechanical allodynia was induced 1, 3, 5 (P < 0.001; F2,15 = 11.20 for both doses) and 7 hours (P < 0.01, F2,15 = 11.20 for 50 ng dose; and P < 0.001, F2,15 = 11.20; for 100 ng dose) after 50 and 100 ng rHMGB1 administration (**Figure 2B**). These findings indicate that rHMGB1 exerts nociceptive potential at the spinal level.

After verifying that both PCT and HMGB1 induced mechanical allodynia, was evaluated the TLR4 and RAGE participation in this process. The TLR4 antagonist LPS-RS reduced PCT-induced mechanical allodynia on day 21 of neuropathic pain. This effect was found 1 and 3h (P < 0.001; F3,20 = 11.90) after 500 ng injection, as well as 3 and 5h (P < 0.001; F3,20 = 11.90) after 1μg injection (**Figure 2C**). Similarly to TLR4, the RAGE antagonist FPS-ZM1 reduced PCT-induced allodynia from 1h (P < 0.01; F4,24 = 16.66) to 3h (P < 0.05; F4,24 = 16.66) after 50 μg injection, while 100 μg injection alleviated the mechanical allodynia from 1h to 7h (P < 0.001, F4,24 = 16.66), and after 24h (P < 0.05, F4,24 = 16.66) compared to control animals (**Figure 2D**).

When TLR4 and RAGE participation in rHMGB1-induced allodynia was evaluated, we verified that the highest effective doses previously reported prevented mechanical allodynia in our PCT-induced neuropathic pain model. FPS-ZM1 (100 µg/5 µL) and LPS-RS (1 µg/5 µL) pretreatment reversed nociception from 1h to 7h (P < 0.001; F3,18 = 15.78 for FPS-ZM1), and after 1h, 3h (P < 0.001; F3,18 = 8.29 for LPS-RS), and 5h (P < 0.01; F3,18 = 8.29 for LPS-RS) of its injections, respectively (**Figures 2E, F**). Additionally, an experiment with TLR4^-/-^ deficient mice was performed to reinforce TLR4 involvement in HMGB1-induced nociception. **Figure 2G** shows that rHMGB1-induced nociception (from 1h to 7h, P < 0.001; F3,18 = 12.30) was completely abolished in TLR4^-/-^ mice. When administered alone, neither the vehicle nor the substances changed the nociceptive threshold compared to baseline values (**Supplementary Figure S1**).

Thus, these results suggest that HMGB1 modulates nociception at the spinal level, and both RAGE and TLR4 participate in this process.

### 3.2 HMGB1 participates in PCT-induced neuropathic pain

**Figure 3:**
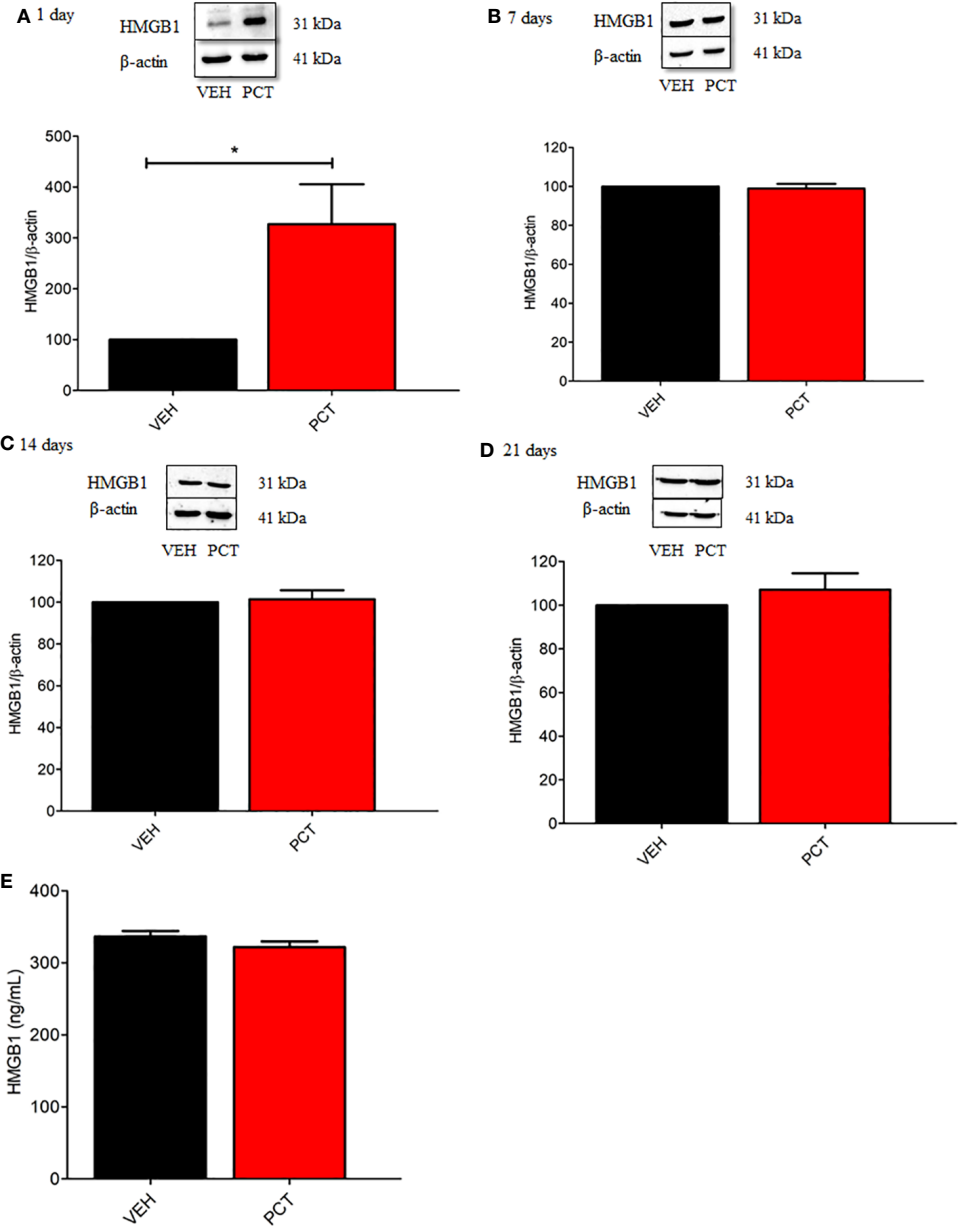
Spinal HMGB1 participates in the early stage of paclitaxel-induced neuropathic pain. Spinal HMGB1 protein levels were evaluated after 1, 7, 14 and 21 days of neuropathic pain **(A–D)**. Furthermore, spinal HMGB1 levels were also assessed after 21 days **(E)**. Data are expressed as the mean ± SEM of 4 per group animals for western blot assay and 6 animals per group for ELISA assay. *P < 0.05 indicates statistical difference compared to animals that received saline (Vehicle, VEH). Student’s t-test. PCT, paclitaxel); VEH.

According to previous evidence, DAMPs are release by death neurons and/or active immune cells after nerve injury, which could bind to receptors promoting inflammatory response and nociception (23, 24). Thus, we evaluated spinal HMGB1 protein levels 1, 7, 14 and 21 days after PCT treatment. Our results found increased protein levels for HMGB1 on the first day only (P < 0.05) (**Figures 3A–D**), suggesting that a single PCT treatment increases HMGB1 spinal release, which can interact with TLR4 and/or RAGE receptors, triggering intracellular cascades that contribute to pain development. Furthermore, HMGB1 spinal levels were similar in all groups (P > 0.05) on day 21 after PCT treatment (**Figure 3E**).

### 3.3 Spinal TLR4, but not RAGE are expressed during PCT-induced neuropathic pain

**Figure 4:**
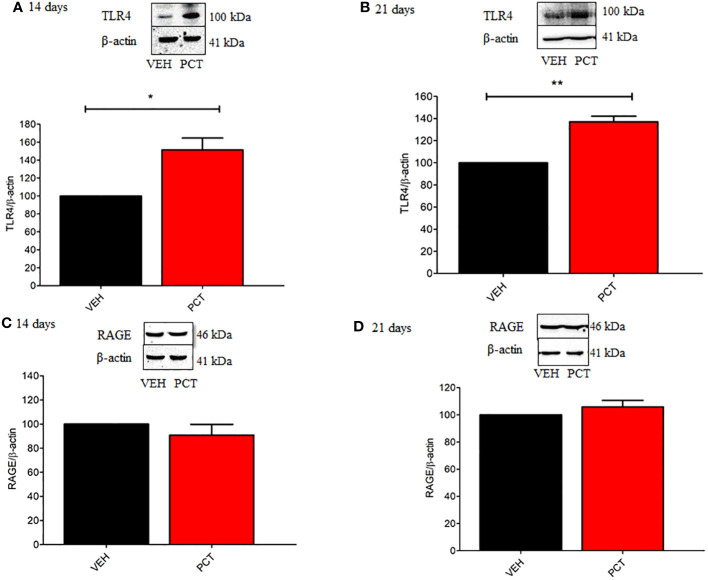
Effect of paclitaxel-induced neuropathic pain on the spinal TLR4 and RAGE protein levels. 8 and 15 days after the end of treatment with paclitaxel (PCT), 14th and 21st day respectively, spinal protein levels of TLR4 **(A, B)** and RAGE **(C, D)** were evaluated. Data are expressed as the mean ± SEM of 4 animals per group. *P < 0.05 and ** P < 0.05 indicate statistical difference compared to animals that received saline (Vehicle: VEH). Student’s t-test.

As behavioral experiments demonstrated TLR4 and RAGE spinal involvement in PCT-induced neuropathic pain, protein levels for both receptors were evaluated. Our results found an over-expression of TLR4 spinal levels on 14 (P < 0.05) and 21 (P < 0.01) days of neuropathy compared to control animals (vehicle-treated mice) (**Figures 4A, B**). Conversely, no difference (P > 0.05) was found for spinal RAGE levels (**Figures 4C, D**).

### 3.4 TLR4 and RAGE involvement on IL-1β and TNF-α spinal upregulation during PCT-induced neuropathic pain

**Figure 5:**
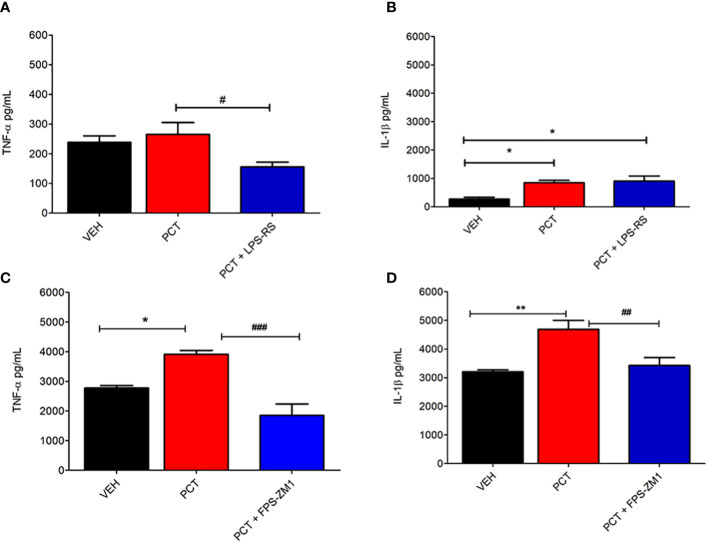
Spinal involvement of TLR4 and RAGE on the pro-inflammatory cytokine levels during paclitaxel-induced neuropathic pain. After 15 days of finishing treatment with paclitaxel (PCT), spinal levels of pro-inflammatory cytokines IL-1β and TNF-α were evaluated in mice pretreated with TLR4 and RAGE antagonists LPS-RS **(A, B)** or FPS-ZM1 **(C, D)**, respectively. Data are expressed as the mean ± SEM of 6 animals per group. *P < 0.05 and **P < 0.01 indicate statistical difference compared to animals that received saline (Vehicle: VEH); #P < 0.05, ##P < 0.01 and ###P < 0.001 indicate statistical difference compared to PCT group. One way ANOVA followed by Bonferroni pos hoc test.

After investigating TLR4 and RAGE participation on PCT-induced neuropathic pain, we evaluated the influence of these receptors on IL-1β and TNF-α spinal levels 21 days after PCT treatment. Our results shown that TNF-α (P < 0.05) but not IL-1β levels was reduced by the TLR4 antagonist LPS-RS (**Figures 5A, B**). In addition, TNF-α (P < 0.01) and IL-1β (P < 0.01) levels were reduced by the RAGE antagonist FPS-ZM1 (**Figures 5C, D**). These findings suggest that TLR4 and RAGE may influence the release of cytokines during neuropathic pain.

### 3.5 Association between glial cells and RAGE and TLR4 during PCT-induced neuropathic pain

**Figure 6:**
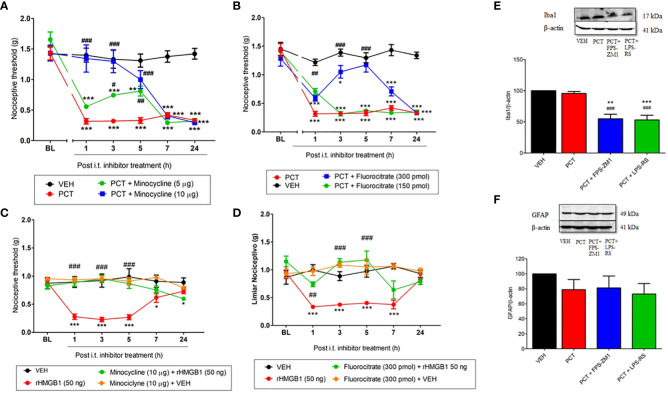
Spinal involvement of TLR4 and RAGE and glial cells during mechanical allodynia induced by paclitaxel or HMGB1. Mechanical allodynia induced after 15 days the end of paclitaxel (PCT) treatment (21st day) or three hours after rHMGB1 i.t. injection was reversed by microglia minocycline **(A, C)** and fluorocitrate **(B, D)** inhibitors, respectively. Furthermore, after 21 days of finishing treatment with PCT, spinal Iba1 **(E)** and GFAP **(F)** levels were evaluated in mice pretreated with TLR4 and RAGE antagonists LPS-RS or FPS-ZM1, respectively. Data are expressed as the mean ± SEM of 4 per group animals for western blot assay and 6 animals per group for behavioral assay. In **(A–D)**, *P < 0.05, **P < 0.01 and ***P < 0.001 indicate statistical difference compared to animals that received saline (Vehicle: VEH); #P < 0.05, ##P< 0.01 and ###P< 0.001 indicate statistical difference compared to PCT or rHMGB1 groups. Two-way ANOVA followed by the Bonferroni pos-hoc test. In **(E, F)**, **P < 0.01 and ***P < 0.001 indicate statistical difference compared to animals that received saline (Vehicle, VEH); ##P < 0.01 and ###P < 0.001 indicate statistical difference compared to PCT. One way ANOVA followed Bonferroni *post hoc* test. BL, baseline latency.

Studies demonstrated that TLR4 and RAGE are expressed in glial cells during neuroinflammation processes (10, 12). Thus, we investigated microglia and astrocytes participation in HMGB1- and PCT-induced mechanical allodynia. The microglia inhibitor minocycline reversed mechanical allodynia 21 days after PCT treatment. This effect was found 3h (P < 0.05; F3,20 = 15.37) and 5h (P < 0.01; F3,20 = 15.37) after 5 μg injection, and from 1h to 5h (P < 0.001; F3,20 = 15.37) after 10 μg injection (**Figure 6A**). Similarly, the astrocytes inhibitor fluorocitrate reduced allodynia 1h (P < 0.01; F3,20 = 13.39) after 150 pmol administration; as well as 3h and 5h (P < 0.001; F3,20 = 13.39) after 300 pmol injection (**Figure 6B**).

When evaluated HMGB1-induced nociception, minocycline (10 μg) pretreatment significantly reduced (P <0.001; F3,20 = 11.21) mechanical allodynia compared to paclitaxel group, and this effect lasted for 5 hours (**Figure 6C**). Similar result was found in the 1st (P < 0.01; F3,20 = 6.73), 3rd and 5th (P < 0.001; F3,20 = 6.73) hour of fluorocitrate (300 pmol) pretreatment (**Figure 6D**).

Once glial involvement was found, its association with TLR4 and RAGE spinal levels was evaluated 21 days after PCT-induced neuropathic pain. Our results demonstrated reduced (P < 0.05) Iba1 spinal levels (a microglia marker) in LPS-RS- and FPS-ZM1-pretreated mice (**Figure 6E**). Regarding astrocytes, no significant difference (P > 0.05) was found in GFAP protein levels (**Figure 6F**).

### 3.6 Spinal p38 MAPK and NF-kB pathways are involved with HMGB1 and PCT-induced nociception

**Figure 7:**
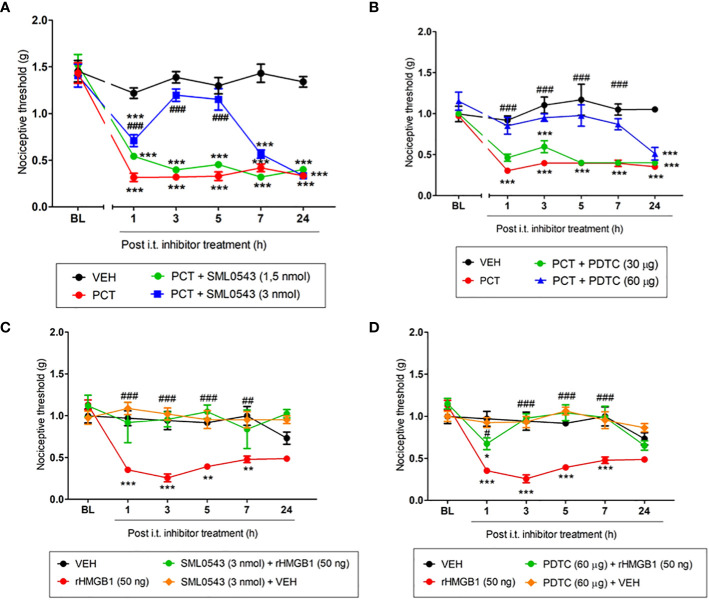
Spinal involvement of p38 MAPK and NF-kB pathway during mechanical allodynia induced by paclitaxel or HMGB1. Mechanical allodynia induced after 15 days the end of paclitaxel (PCT) treatment (21st day) or three hours after rHMGB1 i.t. injection was reversed by specific p38 **(A, C)** and NF-kB **(B, D)** inhibitors, respectively. Data are expressed as the mean ± SEM of 4-6 animals per group. In **(A–D)**, *P < 0.05, **P < 0.01 and ***P < 0.001 indicate statistical difference compared to animals that received saline (Vehicle, VEH); #P < 0.05, ##P< 0.01 and ###P < 0.001 indicate statistical difference compared to PCT or rHMGB1 groups. Two-way ANOVA followed by the Bonferroni pos-hoc test. BL, baseline latency.

Several studies have shown the involvement of the intracellular signaling cascade p38 MAPK/NF-kB after glial cells activation, mainly microglia (25, 26). In this context, specific inhibitors of this pathway (SML0543 and PDTC) were used to evaluate its involvement in PCT-induced neuropathic pain. At 3 pmol, SML0543 reduced (P < 0.001; f3,20 = 14.33) mechanical allodynia from 1h to 5h after injection (**Figure 7A**). A similar effect (P < 0.001; F3,20 = 4.12) was found 1h to 7h after PDTC (60 µg/5 µL) injection (**Figure 7B**).

When evaluating spinal p38 MAPK/NF-kB involvement in HMGB1-induced nociception, mechanical allodynia was reversed from 1h to 5h hour (P < 0.001; F3,18 = 7.36), and 7h hour (P < 0.01; F3,18 = 7.36) after 3 pmol SML0543 i.t. administration (**Figure 7C**). rHMGB1-induced nociception was also attenuated 1h (P < 0.05; F3,18 = 7.36) after treatment with the NF-kB inhibitor PDTC. rHMGB1-induced nociception was completely reversed 3h, 5h and 7h (P < 0.001; F3,18 = 7.36) after PDTC administration (**Figure 7D**). Our results suggest that the p38 MAPK/NF-kB pathway participates spinally in the nociception found both by HMGB1 administration and PCT treatment.

### 3.7 HMGB1 directly activates microglia by leading to the production of TNF-α

**Figure 8:**
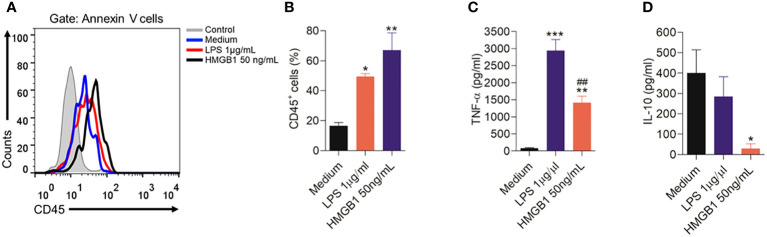
Activation of microglia cells by HMGB1. **(A)** Representative plots showing the cell analysis of the microglia cells after stimulation for 12 hours with HMGB1 or LPS analyzed by flow cytometry, and **(B)** Indicates the CD45 expression after treatment with HMGB1 or LPS. **(C, D)** indicate levels of the cytokines TNF-α and IL-10 in culture medium of microglia stimulated with LPS or HMGB1 or culture medium (control). Data are expressed as the means ± SD. *P < 0.05, **P < 0.01 and ***P < 0.001 indicate statistical difference compared to medium (without treatment); ^##^P< 0.01 indicate statistical difference compared to LPS group.

In order to confirm one of the hypotheses of the present study that HMGB1 can activate microglia, leading to the release of pro-inflammatory cytokines, which may be involved in nociception, we performed some *in vitro* experiments. Thus, we found that after 12 hours of HMGB1 treatment led to a significant (P < 0.01) increase in CD45 expression on the surface of microglia, indicating a heightened state of immune activation in response to HMGB1 (**Figures 8A, B**). The LPS, used as positive control also induced a significant (P < 0.05) microglia activation.

In addition, after HMGB1 treatment a significant (P < 0.01) increase in the pro-inflammatory cytokine TNF-α was found (**Figure 8C**). A similar result was found by LPS treatment. Furthermore, when evaluating the effect of microglia stimulation with HMGB1, it was found that this treatment reduced (P < 0.05) the levels of the anti-inflammatory cytokine IL-10 (**Figure 8D**).

Supporting the *in vivo* results, these results suggest that HMGB1 can activate microglia resulting in the release of TNF-α and the inhibition of IL-10, favoring nociception.

## 4 Discussion

The present study provides the evidence that RAGE and mainly TLR4 are involved in PCT-induced neuropathic pain at the spinal level. In addition, the p38 MAPK/NF-kB pathway and the pro-inflammatory cytokines IL-1β and TNF-α also have potential participation in this process. By using i.t rHMGB1 administration (the main RAGE and TLR4 ligand), we demonstrated that this protein can trigger nociception at the spinal level through the mechanisms previously presented. Finally, our findings indicated that HMGB1/TLR4/p38 MAPK/NF-kB pathway activation and pro-inflammatory cytokines upregulation may be present in microglia, which characterizes a neuroinflammation process that may contribute to nociception induced by PCT.

Our study used the PCT-induced neuropathic pain model, in which we observed a long-lasting reduction in the nociceptive threshold, corroborating previous studies (15, 27). However, we used a protocol with lower PCT administrations. Knowing that PCT promotes peripheral nerves degeneration and leads to DAMPs release, and that HMGB1 is constitutively expressed in nerve cells and can also be released during nerve injury (8), HMGB1-induced spinal nociceptive effect was evaluated. Thus, we observed an important role of this DAMP in spinal nociception in rHMGB1-treated animals and PCT-induced neuropathic pain, particularly in an initial stage that manifest increased HMGB1 spinal levels. Thus, the HMGB1 may be involved in pain modulation at the spinal level. During chemotherapy, HMGB1 may be released, which may participate in the development of neuropathic pain by activating specific receptors. This may be explained considering that HMGB1 takes around 8h to translocate to cytoplasm and 18h to be released in the extracellular space (28, 29). Thus, these aspects are in line with our findings indicating HMGB1 involvement in neuropathic pain after the first day of PCT treatment.

In addition, HMGB1 can interact with receptors expressed on immune cells, such as TLR4 and RAGE, whose activation contributes to neuropathic pain development (30, 31). Furthermore, the present study revealed that TLR4 and RAGE are involved in the nociception induced by spinal rHMGB1 injection or PCT-induced neuropathic pain. Despite behavioral pharmacological experiments demonstrating RAGE involvement during neuropathic pain, tissue levels of this receptor were not altered. We believe that although RAGE expression is not increased, basal levels of this receptor participate in the nociceptive process. Sekiguchi et al. (15) demonstrated RAGE involvement in PCT-induced neuropathic pain. However, the authors reported RAGE activation by HMGB1 released from peripheral macrophages, mainly in the dorsal root ganglion. In this study was found that RAGE blockade reduced Iba1 expression, demonstrating a possible spinal participation of the HMGB1-RAGE-microglia pathway in PCT-induced neuropathic pain. Although astrocytes are involved in nociception found after PCT treatment (6, 7), we did not find changes in GFAP expression, a classic marker of this cell type.

Since the expression of Iba1 and GFAP did not increase during neuropathic pain, another hypothesis would be that both TLR4 and RAGE could be increased in second-order neurons (32, 33), leading to an increase in their excitability and consequently nociception. This hypothesis is reinforced by some studies that demonstrated the expression of TLR4 and RAGE in neurons (34, 35). Furthermore, HMGB1 may also be released from macrophages and sensitize first-order neurons, contributing to the increase in nociceptive impulse (15).

In addition to RAGE, our results indicated that spinal TLR4 is potentially involved with PCT-induced neuropathic pain. Both TLR4 antagonist and TLR4^-/-^ deficiency abolished PCT-mediated mechanical allodynia. Furthermore, TLR4 spinal levels were increased in mice with neuropathic pain. Yan et al. (36) have already demonstrated that intravenous PCT administration increases TLR4 spinal cord levels. Additionally, our results indicated that TLR4 antagonism down-regulates the spinal Iba1 expression, suggesting TLR4 participation in neuropathic pain development in PCT-treated mice via spinal microglia activation. Our data are supported by another study showing decreased TLR4 spinal cord levels after minocycline (a microglia inhibitor) pretreatment in model of neuropathic pain by sciatic nerve chronic constriction (37).

Both RAGE and TLR4 can be activated by HMGB1 via its passive release after sensory neurons damage, or by active secretion from immune cells during pathological conditions (38). During active secretion, macrophages accumulate in the DRG and release HMGB1. Thus, RAGE is activated facilitating the nociceptive impulse (15, 39). Furthermore, RAGE activation in DRG sensory neurons during neuropathic pain occurs via HMGB1 in thiol redox state (6). HMGB1 redox state is crucial for receptor binding. HMGB1 has 3 cysteines that can be oxidized or reduced. When fully oxidized it loses its pro-inflammatory activity; however, if they are reduced, they can function as chemokines (40). Interestingly, if only residues c23 and c45 were oxidized, they could lead HMGB1 to an intramolecular disulfide bond (dsHMGB1), causing it to have the ability to bind to TLR4 (40). RAGE can bind to all HMGB1 isoforms (40).

Although TLR4 and RAGE are activated during chemotherapy-induced neuropathic pain, as demonstrated previously, TLR4 activation via HMGB1 occurs when HMGB1 exhibits disulfide redox state, which are related to the oxidative environment caused by reactive oxygen species release from mitochondrial damage. This process triggers cytokines production in a similar pathway to that found in the present study, since TNF-α levels and nociception are attenuated by TLR4 antagonism (28, 41).

Our data also demonstrated a spinal involvement of the p38 MAPK/NF-kB pathways during HMGB1- and PCT-induced nociception. Thus, mechanical allodynia was reversed by inhibitors of this signaling pathway. A recent study indicated that this pathway is activated in the dorsal ganglia root, participating in PCT-induced neuropathic pain in mice (42). Another study found that HMGB1, NF-kB, TNF-α and IL-1β expression is upregulated in the anterior cingulate cortex during inflammatory pain, which is an important brain area involved with pain regulation. In addition, HMGB1 inhibition decreased TLR4/NF-kB and proinflammatory cytokine levels (26). Several evidence have demonstrated that pro-inflammatory cytokines, especially TNF and IL1, are produced by microglia and spinal astrocytes, being responsible for the central sensitization of pain, via activation of specific receptors in second-order neurons, leading to an increase in the function of receptors for AMPA and NMDA, enhancing the excitatory synapse (26, 43–45). Taken together, these findings support the results of this study, reinforcing the proposition that HMGB1 participates in TLR4 spinal activation, triggering the production of inflammatory mediators involved with the nociception during PCT-induced neuropathic pain.

It is important to highlight that the results found in the present study were in male mice, and future studies could be carried out in females to evaluate the similarity of the proposed mechanism. Studies have demonstrated an increase in mechanical allodynia and thermal hyperalgesia in female mice subjected to different models of neuropathic pain (46–48), including chemotherapy-induced neuropathic pain (49). Hormonal, sensory, affective and cognitive factors are suggested to participate in these differences in the nociceptive response found in females (50). Furthermore, these factors may directly influence the release of mediators and neurotransmitters involved in this response. Supporting the hypothesis that mechanisms may be different in females than in males, one study found that intrathecal administration of HMBG1 promoted greater increases in spinal levels of pro-inflammatory cytokines and chemokines in males compared to females (51). Furthermore, these authors have demonstrated that spinal involvement of microglia during pain is restricted to males.

**Figure 9:**
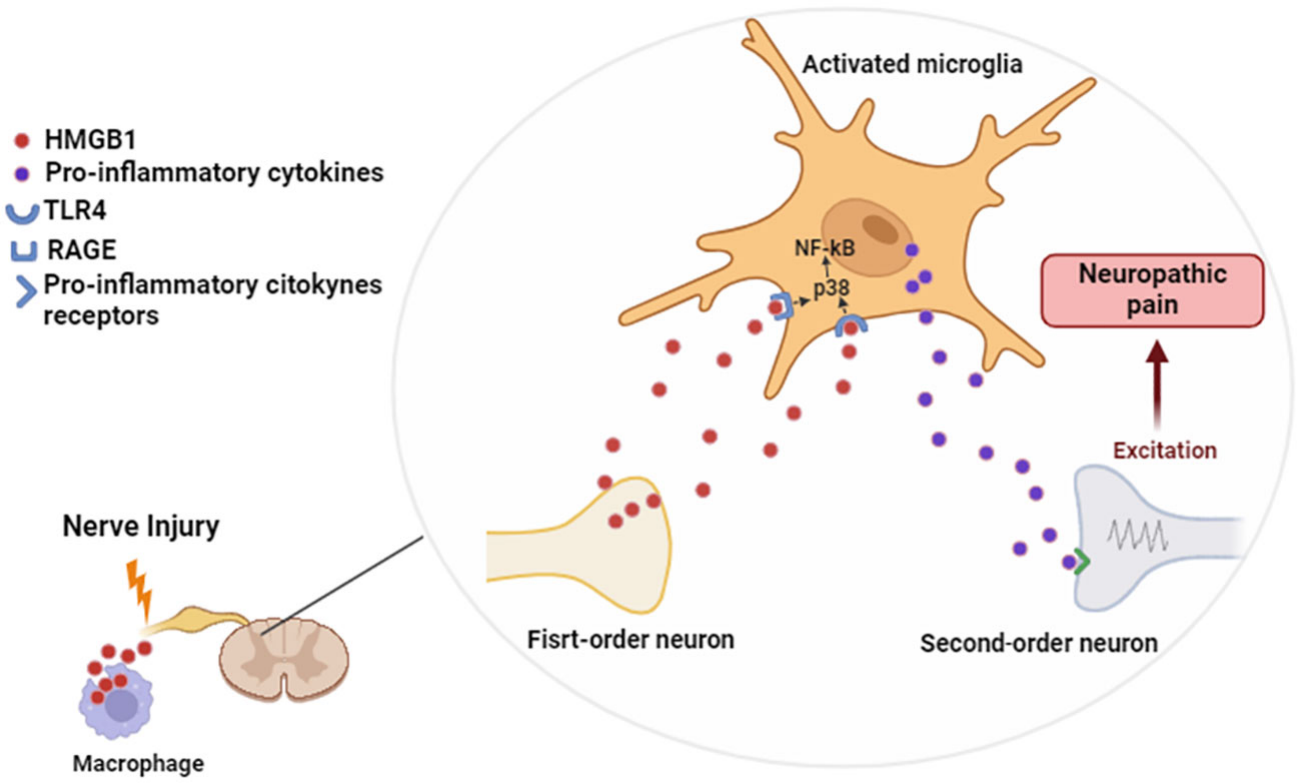
Proposed mechanism of spinal action of HMGB1 during neuropathic pain. Figure created using Biorender.

We conclude that HMGB1 is a key protein during the nociception transmission at the spinal level, mainly during PCT-induced neuropathic pain. Furthermore, we suggested that the release of this DAMP from neurons or macrophages occurs in the first days after PCT administration, which may lead to a later RAGE, TLR4 and microglia activation. This process triggers p38 MAPK and NF-kB intracellular activation, stimulating the production of cytokines that favor neuropathic pain maintenance (**Figure 9**). These data may be useful for further translational studies that aim to delimit innovative strategies to prevent neuropathic pain development at the beginning of chemotherapy treatment.

## Data availability statement

The raw data supporting the conclusions of this article will be made available by the authors, without undue reservation.

## Ethics statement

The animal studies were approved by Ethics Committee for Animal Research approved this study (protocol number 52/2018, UNIFAL). The studies were conducted in accordance with the local legislation and institutional requirements. Written informed consent was obtained from the owners for the participation of their animals in this study.

## Author contributions

TM: Conceptualization, Data curation, Investigation, Methodology, Writing – original draft. FV: Investigation, Methodology, Writing – original draft. AB: Investigation, Methodology, Writing – original draft. EN: Investigation, Methodology, Writing – original draft. AK: Data curation, Investigation, Methodology, Writing – original draft. GG: Funding acquisition, Project administration, Supervision, Writing – original draft.

## Supplementary material

Supplementary Figure 1 Evaluation of the effect of i.t. injection of the LPS-RS, SML0543, FPS-ZM1, PDTC, fluorocitrate (FC), minocycline and vehicles on the nociceptive threshold. Data are expressed as the mean ± SEM of 5 animals per group. Two-way ANOVA followed by the Bonferroni test (factors: time and treatment). BL, baseline latency.
