# Supplementary figures and images for: Spinal HMGB1 participates in the early stages of paclitaxel-induced neuropathic pain via microglial TLR4 and RAGE activation
Source: Front Immunol. 2024 Feb 7;15:1303937. doi: 10.3389/fimmu.2024.1303937 (PMC10879568; doi:10.3389/fimmu.2024.1303937)

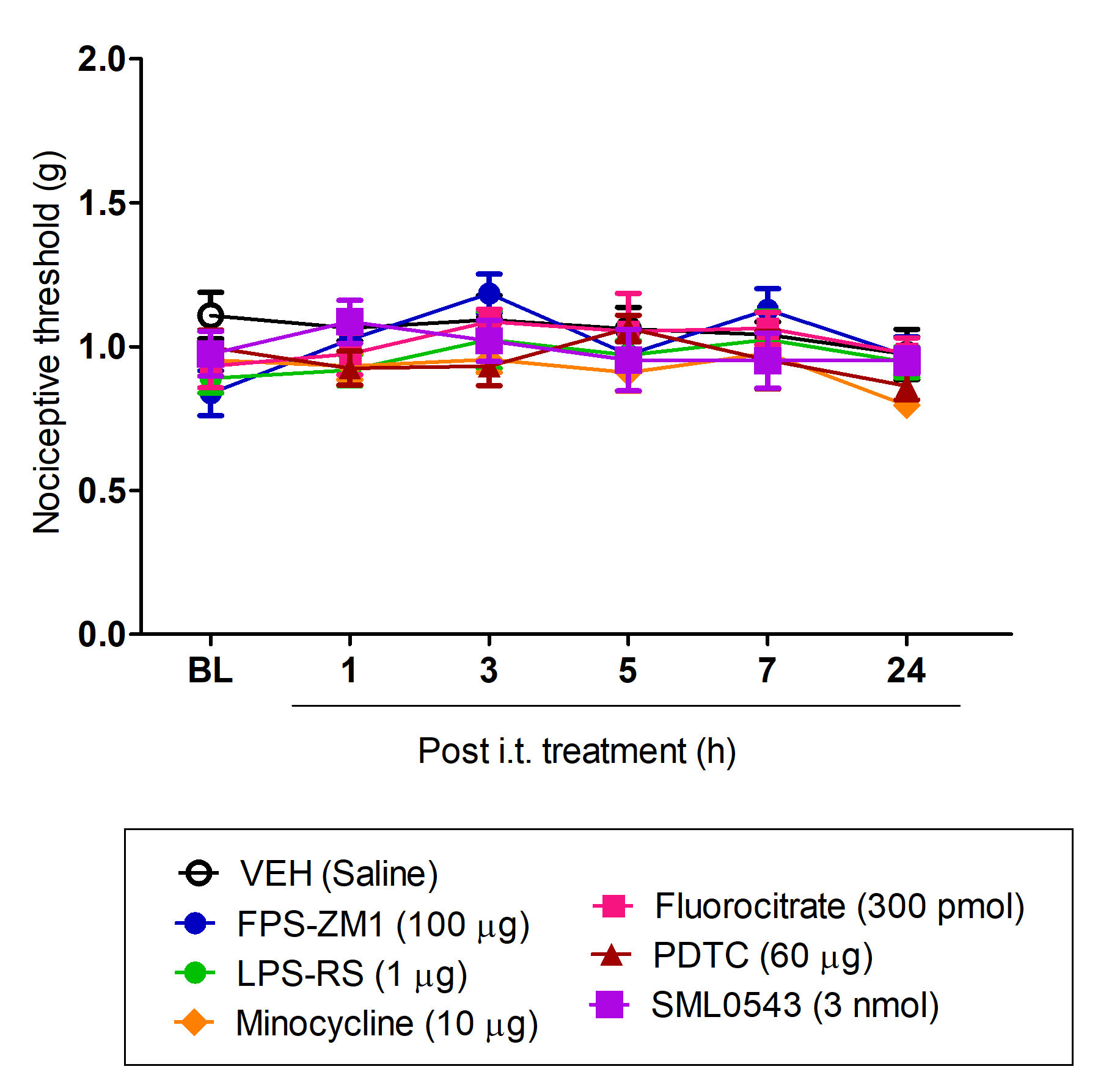

Supplement: Supplementary Figure 1 — Evaluation of the effect of i.t. injection of the LPS-RS, SML0543, FPS-ZM1, PDTC, fluorocitrate (FC), minocycline and vehicles on the nociceptive threshold. Data are expressed as the mean ± SEM of 5 animals per group. Two-way ANOVA followed by the Bonferroni test (factors: time and treatment). BL, baseline latency. [file Image_1.jpeg]
